# Targeting vascular normalization: a promising strategy to improve immune–vascular crosstalk in cancer immunotherapy

**DOI:** 10.3389/fimmu.2023.1291530

**Published:** 2023-12-15

**Authors:** Cheng Qian, Chaoqun Liu, Weiwei Liu, Rui Zhou, Liang Zhao

**Affiliations:** ^1^ Department of Pathology, Nanfang Hospital, Southern Medical University, Guangzhou, China; ^2^ Department of Pathology & Guangdong Province Key Laboratory of Molecular Tumor Pathology, School of Basic Medical Sciences, Southern Medical University, Guangzhou, China

**Keywords:** vascular normalization, cancer immunotherapy, TME (tumor microenvironment), angiogenic, anti-angiogenic

## Abstract

Blood vessels are a key target for cancer therapy. Compared with the healthy vasculature, tumor blood vessels are extremely immature, highly permeable, and deficient in pericytes. The aberrantly vascularized tumor microenvironment is characterized by hypoxia, low pH, high interstitial pressure, and immunosuppression. The efficacy of chemotherapy, radiotherapy, and immunotherapy is affected by abnormal blood vessels. Some anti-angiogenic drugs show vascular normalization effects in addition to targeting angiogenesis. Reversing the abnormal state of blood vessels creates a normal microenvironment, essential for various cancer treatments, specifically immunotherapy. In addition, immune cells and molecules are involved in the regulation of angiogenesis. Therefore, combining vascular normalization with immunotherapy may increase the efficacy of immunotherapy and reduce the risk of adverse reactions. In this review, we discussed the structure, function, and formation of abnormal vessels. In addition, we elaborated on the role of the immunosuppressive microenvironment in the formation of abnormal vessels. Finally, we described the clinical challenges associated with the combination of immunotherapy with vascular normalization, and highlighted future research directions in this therapeutic area.

## 1 Introduction

Angiogenesis is an important hallmark of malignant tumors (1). Tumor angiogenesis refers to the process of neovascularization during tumor growth. It is induced by high concentrations of pro-angiogenic factors secreted by tumor cells to obtain an adequate supply of blood and nutrients. The concept of vascular normalization—proposed by Rakesh K. Jain in 2001 (2)—refers to the transformation of tumor blood vessels and their microenvironment from an abnormal structural and functional state to a normal one. Consequently, tumor cells are freed from hypoxia and the tumor microenvironment is reprogrammed.

Tumor blood vessels are different in structure and function from the normal tissues. The abnormal blood vessels of tumors are dilated, tortuous, disordered, immature, and impermeable and lack connections between parietal cells (3). The production of numerous abnormal blood vessels has deleterious effects that promote tumor development. Anti-angiogenic therapy deprives the tumor of oxygen and nutrients to induce necrosis. However, clinical use of anti-angiogenic drugs has led to unexpected outcomes, such as chemotherapy resistance and increased propensity to metastasis. This may be attributed to the structural changes in immature newly developed vasculature and its resulting dysfunction after the destruction of a large number of blood vessels by anti-angiogenic therapy (2, 4–6). Therefore, the focus of vascular research in oncology has shifted from understanding only anti-angiogenesis to normalizing tumor blood vessels. A window of vascular normalization can be achieved transiently during the initial days of anti-angiogenesis therapy. Normalized blood vessels can reverse drug resistance and immune suppression in patients with cancer (5). Therefore, the perfect drug dosage, exposure time, and usage range to attain the vascular normalization window are currently being explored to develop effective cancer therapeutic strategies.

A comprehensive analysis of the tumor microenvironment (TME) has revealed that tumor blood vessels cannot be understood from a single perspective. They have heterogeneous structure, function, and organization, and immune cells, cytokines, and extracellular matrix (ECM) interact with them (7, 8).

Immunotherapy has revolutionized the treatment of tumors by improving immune function in the TME. Immunotherapies, such as the chimeric antigen receptor (CAR)-T-cell therapy, enhance the function of the effector T cells. CARs are designed to create T-cell clones that can specifically recognize and clear tumors containing specific antigens or mutated proteins. Other approaches, such as immune checkpoint inhibitor therapies, relieve the immune suppression of T cells (9, 10). These inhibitors are designed to target inhibitory ligand–receptor interactions between T cells and other cells in the TME. Representative examples include monoclonal antibodies targeting cytotoxic T-lymphocyte-associated protein-4 (CTLA-4) and antibodies blocking programmed cell death-1 (PD-1)/programmed cell death ligand-1 (PD-L1). However, all patients do not respond well to immunotherapy, and the overall clinical response rate of tumor immunotherapy needs to be improved (10). The TME is characterized by hypoxia, low pH, and high interstitial fluid pressure. Blood vessels are an important component of the TME and substantially contribute to the formation of abnormal TME, ultimately affecting the response of patients to immunotherapy (7).

Normal blood vessels help immune cells adhere to and penetrate the tumors. Recently, some authors have suggested that improving immune function can promote vascular normalization (11, 12). Therefore, combining vascular normalization with immunotherapy may be a feasible approach to improve treatment outcomes. Although several authors have evaluated the combination of vascular normalization and immunotherapy in clinical studies, the dosage and timing of anti-angiogenic drugs and immunotherapy are still controversial. Several clinically relevant biomarkers are available to quantify the extent of vascular normalization. However, the mechanism underlying the vascular normalization-mediated cancer therapies needs to be further studied.

In this review, we focused on the interplay between vascular normalization and immunotherapy from the perspective of the TME and summarized the clinical effects of combining them. In addition, we elaborated on the adverse consequences of abnormal blood vessels on tumor treatment and approaches that can normalize tumor blood vessels. Moreover, combining immunotherapy with drugs for vascular normalization, the problems caused by tumor heterogeneity, and strategies to overcome these obstacles were also discussed.

## 2 Normalization of tumor vasculature

Tumor blood vessels are structurally and functionally different from those in the normal tissues. They are dilated, tortuous, disordered, immature, and impermeable and lack connections between parietal cells (3). These structural abnormalities lead to hypoxia and hypoperfusion in the TME.

### 2.1 Abnormal structure and function of tumor blood vessels

Pericytes and endothelial cells (ECs) are proximal in the normal tissues and communicate through intercellular contacts and secreting specific molecules. This communication results in an orderly arrangement of ECs and the formation of an effective, organized, and mature vascular network (13). However, the connections among perivascular cells, basement membranes, and ECs are impaired in tumors. Tumor-associated pericytes show abnormal morphology and are low in density compared with normal tissue pericytes.​The weakening of these connections renders the vessels impermeable. Consequently, blood leaks into the stroma, causing a hypoperfusion in the tumor tissues. In addition, the increased interstitial pressure due to leakage, the compression of the blood vessels by tumors, and the dysfunction of the lymphatic network aggravate the hypoperfusion in the tumor tissues.

### 2.2 Tumor angiogenesis

Angiogenesis refers to the formation of new blood vessel branches in an existing blood vessel network. The two phenotypes of endothelial cells, tip and stalk cells, are involved in the initial formation of blood vessels. Tip cells participate in angiogenic sprout formation, and endothelial cells contribute to lumen formation through a pinocytotic process. Finally, new vessels undergo maturation through pericyte recruitment, basement membrane formation, and stronger endothelial cell connections. Several unique neovascularization patterns exist in the tumor tissues, including vascular co-option and vascular mimicry. Vascular co-option is a non-angiogenic process in which tumor cells use pre-existing blood vessels to support tumor growth, survival, and metastasis. This process is independent of vascular endothelial growth factor (VEGF) and occurs in the absence of typical angiogenic processes. Notably, VEGF inhibitors do not the inhibit progression and invasion of such tumors. Vasculogenic mimicry is the formation of a “vessel-like” structure without endothelial cells. This structure is characterized by a fluid-conducting tube with a lumen that is capable of providing oxygen and nutrients and eliminating cellular waste (3, 14). Overall, abnormal tumor vascular function and architecture lead to malignant outcomes.

### 2.3 Molecular mechanisms of abnormal tumor angiogenesis

The physiologic balance between anti-angiogenic and pro-angiogenic factors is disrupted in the tumor tissues. High concentrations of pro-angiogenic factors, such as VEGF, angiopoietin 2 (ANG2), and platelet-derived growth factor (PDGF), are present in the TME (15) of various cancers, such as non-small cell lung cancer, gastric cancer, colorectal cancer, and glioma (16–20). In addition, hypoxia is responsible for tumor angiogenesis. Prolyl hydroxylase domain protein 2 is inactive under hypoxic conditions and cannot use oxygen to degrade hypoxia-inducible factor (HIF). Notably, HIF is ubiquitinated by the Von Hippel–Lindau complex and degraded by proteasomes. Hypoxic conditions inhibit HIF-α degradation, leading to the dimerization of accumulated HIF-α and HIF-β. These dimers bind to the hypoxia response elements to activate the target genes of HIF. Finally, pro-angiogenic factors, such as VEGFA, transforming growth factor (TGF)-β, and PDGF-B are released (21–23). These factors increase vascular permeability and promote endothelial cell proliferation, sprouting, migration, adhesion, and tube formation. Several authors have determined the downstream signaling of VEGF receptor (VEGFR) after the binding of VEGF. Some known downstream pathways of VEGF include the Ras/MAPK pathway regulating cell proliferation and gene expression, the FAK/paxillin pathway involved in cytoskeleton rearrangement, the PI3K/AKT pathway regulating cell survival, and the PLC-γ pathway controlling vascular permeability (19, 24). In addition, the Hippo pathway plays a role in vascular cell migration and VEGFR-induced angiogenesis. The activation of VEGFR by VEGF triggers the PI3K/MAPK signaling, which subsequently inhibits LATS (a key component of the pathway) and then activates the Hippo effectors, including Yes-associated protein and transcriptional co-activator with PDZ-binding motif (25–27).

Early growth response-1 (EGR-1) is an important upstream transcription factor that regulates cell proliferation and differentiation and plays an essential role in tumor angiogenesis (28, 29). EGR-1 is a common intermediate of VEGF and fibroblast growth factor (FGF) in regulating cell function (30). Yu, J. et al. revealed a specific mechanism by which EGR-1 leads to tumor angiogenesis. The capacity to promote angiogenesis was attenuated in the nuclear PD-L1-deficient cells *in vivo* and *in vitro*. Mechanistically, nuclear PD-L1 facilitated the binding of p-STAT3 to the EGR-1 promoter, resulting in the activation of EGR-1-mediated angiogenesis. Moreover, a new anti-angiogenic strategy has been developed to block the nuclear translocation of PD-L1 by inhibiting histone deacetylase 2 and restoring PD-L1 acetylation levels (31).

## 3 Consequences of abnormal tumor vasculature

Abnormal tumor vasculature can promote tumor metastasis and affect the prognosis in patients (32). In addition, abnormal tumor angiogenesis decreases the efficacy of antitumor therapies, including radiotherapy, chemotherapy, and immunotherapy (3).

### 3.1 Immunosuppression

**Figure 1:**
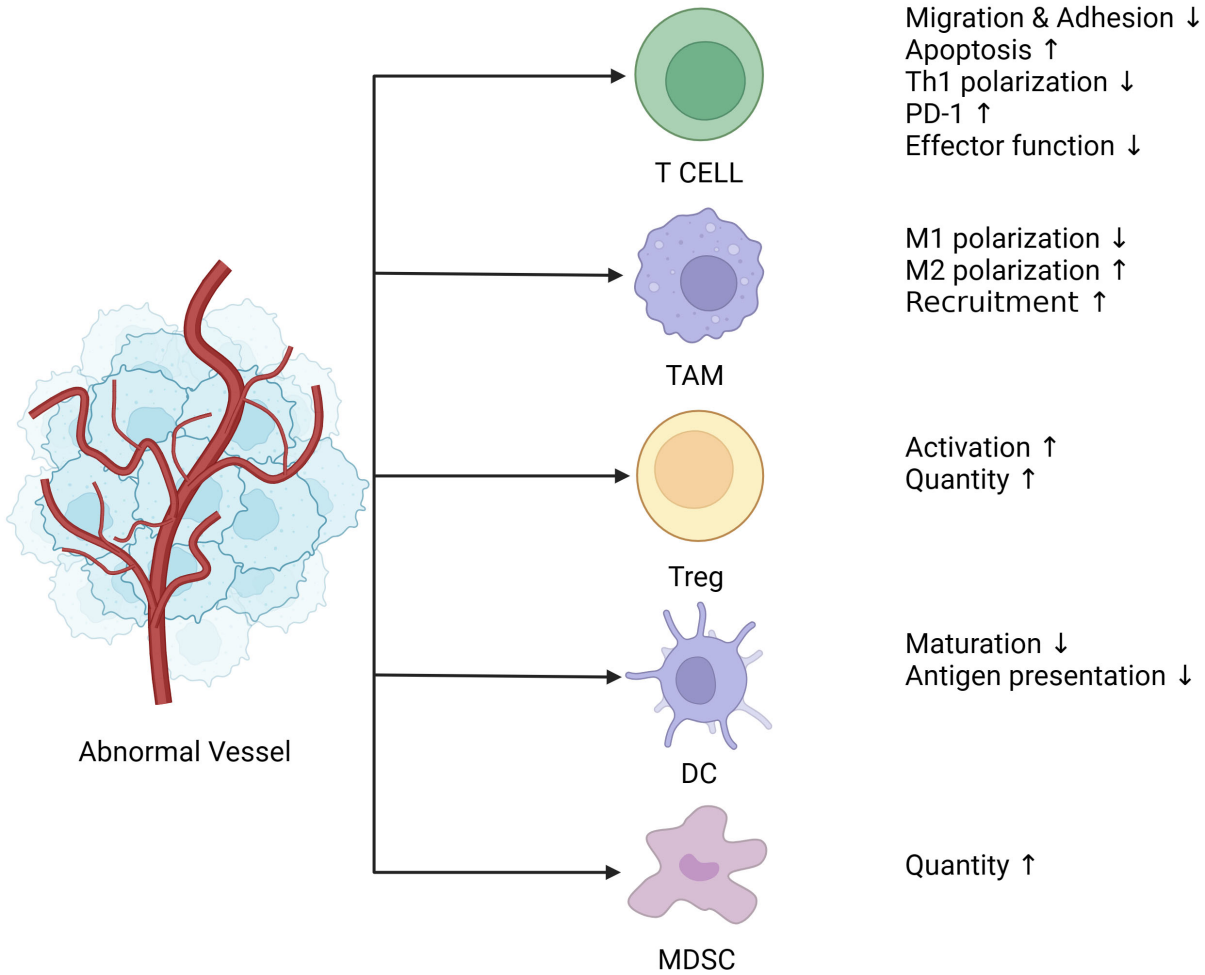
Abnormal blood vessels lead to immunosuppression. Tumor blood vessels are characterized by uneven distribution, tortuosity, clutter, hypertonicity, lack of pericyte coverage, and their ability to deliver oxygen and nutrients is also compromised, followed by a tumor microenvironment of hypoxia, low Ph, and high interstitial pressure. Hypoxia leads to the release of angiogenic factors such as VEGF and ANG (Angiogenin), which together with endothelial cells create an immunosuppressive microenvironment. Endothelial cells are less responsive to inflammatory stimuli, and the expression of adhesion molecules is reduced. The adhesion and migration function of immune cells such as T cells is decreased, which directly contributes to the reduction of T cell infiltration in tumors. In addition, PD-1(Programmed death-1) expression on the surface of T cells was up-regulated, and activation of T cells and polarization of CD4+T cells to Th1 cells were suppressed. Moreover, TAM is polarized to M2 type under the action of VEGF, which is an immunosuppressive phenotype and one of the major contributors to the immunosuppressive microenvironment. The release of VEGF also recruits a large number of MDSC, Treg cells, both of which are immunosuppressive cells, and Treg cells have proliferation and functional advantages under hypoxia and low Ph conditions. Finally, DCs (Dendritic cells), most of which are immature DCs infiltrated in tumors under hypoxia, have impaired presentation function. (The figure was designed in Biorender.com).

Antitumor immunity is a sequential process. Chen and Mellman proposed the “cancer immune cycle” and summarized it into seven stages (33). First, dendritic cells (DCs) capture the tumor antigen and present the captured antigen to T cells, consequently initiating effector T-cell responses. Finally, activated effector T cells infiltrate the tumor bed, recognize tumor antigens, bind to tumor cells, and kill their target cells. The killed tumor cells release additional tumor-associated antigens that increase the intensity of the immune response. The abnormal formation of tumor blood vessels affects tumor immunity at various stages through several physical and biochemical mechanisms (**Figure 1**).

#### 3.1.1 Effects on trafficking and infiltration of immune effector cells

The immune landscape within the TME is of three major types, namely immune infiltration, immune exclusion, and immune silent (34, 35). The “immune infiltrated” tumors are the tumors containing abundant infiltrated immune cells, such as cytotoxic T lymphoctyes (CTLs), which induce an active immune response. “Immune excluded” tumors have T cells only at their periphery, which do not infiltrate the TME. “Immune silent” tumors are the ones with little or no immune infiltration. Immune cells need to adhere and penetrate to reach the tumor tissues, and a normal vascular network is essential for this process (35). Tumor vascular tissue is structurally and functionally different from the normal vascular tissue. The heterogeneous vascular network results in hypoperfusion within tumors that have few immune cells to allocate. Moreover, immature tumor vascular tissue is prone to leakage due to insufficient connectivity between endothelial cells and the insufficient encapsulation of basement membranes and pericytes (36). Consequently, a large amount of fluid enters the interstitial space from blood vessels. Consequently, a high-pressure state is created in the interstitium, prohibits the oxygen and nutrients from entering the tumor tissues (37), resulting in a hypoxic environment. The hypoxic microenvironment attracts and sequesters tumor-associated myeloid cells and CTLs (cytotoxic T lymphocytes), resulting in the accumulation of tumor-associated myeloid cells in the hypoxic region. These cells are reprogrammed to an immunosuppressive state, limiting the efficacy of immunotherapy (38). In addition, the hypoxic environment induces the production of pro-angiogenic factors, such as VEGF, which aggravate vascular abnormality. Dorsal meningeal lymphatic vessels (MLVs) undergo extensive remodeling in mice with intracranial gliomas or metastatic melanomas. Disruption of dorsal MLVs impaired intratumor fluid drainage, disseminated brain tumor cells to deep cervical lymph nodes (dCLNs), decreased the DC trafficking from intracranial tumor tissues to dCLNs, and weakened the effect of anti-PD-1/CTLA-4 checkpoint therapy (39).

The anergy of ECs has been proposed as one of the reasons for the immunosuppression caused by abnormal blood vessels. The EC anergy results in a reduced leukocyte–vessel wall interaction by stimulating the expression of immunosuppressive molecules in abnormal tumor vessels (40). Leukocytes do not adhere to ECs without inflammatory stimulus. AP1 and NF-κB signaling activates the expression of endothelial adhesion molecules, including E-selectin, intercellular cell adhesion molecule (ICAM-1), and vascular cell adhesion molecule-1 in the presence of inflammation, which allows leukocytes to migrate across the endothelium into the extravascular space (41, 42). ECs in abnormal blood vessels, stimulated by various pro-angiogenic factors secreted by tumor tissues, exhibit a state of non-responsiveness to inflammatory stimuli. These non-responsive ECs fail to enhance the expression of endothelial adhesion molecules to stimulate the adhesion and penetration of immune cells (43, 44). Tumor ECs express immunosuppressive molecules, such as carbohydrate-binding protein galectin 1, FAS-L, TIM-3, PD-L1, and indoleamine-2,3-dioxygenase, to create an immunosuppressive TME by inducing apoptosis of activated T cells, inhibiting the activation and polarization of CD4^+^ Th cells to Th1 cells, promoting the activation of Treg cells, and enhancing the immunosuppressive effect of CD8^+^ T cells (45–48). Interestingly, tumor endothelial anergy can be overcome by sunitinib or bevacizumab treatment in renal cell carcinoma (RCC). In addition, a correlation between increased immune cell infiltration and ICAM-1 expression was observed after VEGF-targeted therapy (49).

#### 3.1.2 Role of angiogenic factors in the TME

Angiogenic factors not only create an abnormal tumor vascular network but also contribute to the immunosuppressed TME. The angiogenic programming in tumor tissues is a multidimensional process that regulates cancer cells, tumor-associated stromal cells, and bioactive products, including cytokines and growth factors, ECM, and secreted microvesicles (50).

Abnormal tumor blood vessels often create a hypoxic TME, which affects tumor immunity by inducing the overexpression of angiogenic factors. Several major angiogenic factors play important roles in immunosuppression.

VEGF The VEGF family of proteins comprises VEGFA, VEGFB, VEGFC, VEGFD, and VEGFE (encoded by viruses) and the pro-angiogenic molecule placental growth factor (PGF/PlGF) (24). VEGF has an immunosuppressive effect. Mice exposed to pathologic VEGF concentrations showed severe thymic atrophy, characterized by a significant reduction in CD4^+^/CD8^+^ thymocytes. Specifically, VEGF, at pathologic concentrations, interfered with the development of early hematopoietic progenitor T cells to block their differentiation and migration, leading to tumor-related immunodeficiency (51). Deng, H. et al. analyzed the Kaplan–Meier Plotter database and found that high expression of VEGFA in patients with progressive hepatocellular carcinoma (HCC) is associated with a poor prognosis (52). VEGF can promote PD-1 expression on CD8^+^ T cells. However, both the number and function of CD8^+^ T cells are significantly increased after anti-VEGF therapy (53). VEGFA acts on VEGFR2 and increases the expression levels of TIM-3, CTLA-4, and Lag-3 on CD8^+^ T cells in a dose-dependent manner, and the expression levels of these molecules correlates with the level of T-cell exhaustion (54). Moreover, VEGF recruits numerous immunosuppressive cells, such as Tregs, myeloid-derived suppressor cells (MDSCs), and M2 macrophages, and inhibits the maturation of DCs. VEGF can promote the polarization of macrophages into M2 immunosuppressive subtype. Moreover, VEGF-induced hypoxia and a low pH microenvironment cause immunosuppression (55–57). VEGF indirectly decreases tumor necrosis factor (TNF)-α-induced lymphocyte adhesion and the expression of several inflammation-related genes, including cytokine–cytokine receptor interaction-related genes (CXCL10, CXCL11, CSF2, and FLT4) and the p38 MAPK pathway-related genes (DUSP4, IL1R1, and MEF2C) (58). In addition, exposure of ECs to pro-angiogenic factors induces a state of anergy, in which they lose the ability to respond to inflammatory cytokines and upregulate the expression of endothelial adhesion molecules (59).

TGF-β plays a dual role in tumor development. TGF-β, as a tumor suppressor, inhibits cell growth and induces apoptosis of precancerous cells. However, tumor-derived TGF-β induces tumorigenic and prometastatic responses including the formation of an immunosuppressive TME in cancer cell clones with inactivated TGF-β pathway (60). TGF-β plays a role as a pro-angiogenic factor in various diseases, including osteoarthritis, and cancer (61, 62). Leucine-rich alpha-2-glycoprotein 1 had a mitogenic effect on ECs and promoted angiogenesis in the presence of TGF-β1 (63). In addition, TGF-β stimulates the synthesis of PDGF-B by ECs and promotes the synthesis of VEGF by non-ECs during the healing phase of immune injury, suggesting that it upregulates the expression of other cytokines to promote angiogenesis (64). TGF-β is a major component of tumor-derived small extracellular vesicles in cancer patients. These vesicles stimulated macrophage chemotaxis and reprogrammed primary human macrophages to a pro-angiogenic phenotype characterized by the upregulation of pro-angiogenic factors and functions (65). TGF-β can significantly inhibit CTL activity (66) and also promote tumor growth independent of CD4^+^ T cells, interferon (IFN)-γ, and CTLs. Targeting the TGF-β pathway in CD4^+^ T cells may inhibit tumor growth by remodeling and normalizing the tumor vascular network. Further study revealed that Interleukin (IL)-4 secreted by Th2 cells plays a vital role in reprogramming the TME. Therefore, TGF-β not only inhibits the function of CTLs but also influences the type II immunity against cancer (67). Moreover, TGF-β influences the activation of macrophages. For example, phosphatidylinositol-binding protein TIPE1 can promote alternative macrophage activation and tumor progression through the PIP3/Akt/TGF-β axis (68).

ANG2 is involved in angiogenesis at the early stages and is linked to tumor immunosuppression. ANG2 was upregulated in both humans and mice in a study of liver metastases from pancreatic neuroendocrine tumors. This observation coincided with poor T-cell infiltration, suggesting an immunosuppressive TME (69). In addition, TIE2-expressing monocytes/macrophages have a tendency to polarize toward the M2 phenotype in the presence of ANG2 in a mouse model, playing an important role in the emergence of immunosuppressive microenvironment (70). ANG2 restricts the antitumor function of monocytes by inhibiting the secretion of TNF-α (71). ANG1 stimulates the binding of pericytes and vascular smooth muscle cells to ECs, thereby stabilizing newly formed blood vessels (72). However, the role of ANG1 in tumor development is controversial. ANG1 promoted colorectal tumor metastasis and growth. In addition, it upregulated carboxypeptidase A4 to promote tumor cell proliferation in triple-negative breast cancer (73, 74). Therefore, further research is required to explore the role of ANG in cancer.

PDGF-BB modulates tumor angiogenesis by inducing erythropoietin production in stromal cells (75). It can regulate various immune cells to shape the immunosuppressive TME. PDGF-BB mediated the infiltration of M2-phenotype tumor-associated macrophages (TAM) into tumor tissue by inducing pericyte- and fibroblast-derived IL-33 in a mouse tumor xenograft model (76). This growth factor enhances IL-4-induced STAT6 activation, which promotes tumor growth through the expansion of MDSCs and inhibition of cytotoxic T-cell response (77). MDSCs expressing PDGF-BB are recruited by CXCL17 in breast cancer cells and facilitate lung metastasis (78). Interestingly, the concentration of PDGF-BB can help predict the fraction of monocytic MDSCs in the peripheral blood of patients with colorectal cancer (CRC) (79). Therefore, PDGF-BB has a diagnostic value in clinical applications.

### 3.2 Resistance to cancer therapy

​Abnormal tumor vasculature can induce resistance to various cancer treatments, which can adversely affect the prognosis of cancer patients (4). Drug resistance due to abnormal vascular networks has three main aspects. First, the low coverage rate of pericytes and the destruction of blood–tumor barrier aggravates hypoxia in the TME and leads to the accumulation of chemotherapeutic drugs in the tumor stroma (80). Pericytes promoted DNA damage repair in glioblastoma cells residing in perivascular niches, ultimately inducing temozolomide chemoresistance. The large amount of CCL5 secreted by pericytes can bind to CCR5 on glioblastoma cells to activate the DNA-dependent protein kinase catalytic subunit-mediated DNA damage repair after temozolomide treatment (81). Moreover, abnormal vascular networks with pericyte proliferation can physically affect the delivery of antitumor drugs (82). Second, tumor vasculature contributes to drug resistance. Chemotherapy-induced IL-8 secretion in tumor tissues increases the expression of ATP-binding cassette subfamily B member 1 transporter on tumor ECs, which counteracts the therapeutic effects of taxol (83). Alteration in vascular morphogenesis is a hallmark of anti-angiogenesis-resistant tumor vessels. EphB4 overexpression leads to vascular resistance by altering vascular morphogenesis and pericyte coverage in experimental SF126 glioma models (84). Tumor vascularization can occur through vascular co-option rather than angiogenesis. This type of tumor vascularization contributes to the resistance to bevacizumab therapy (85). The occurrence of vascular co-option is related to the increased expression of fibroblast activation protein-α in co-opted hepatic stellate cells in the bevacizumab-resistant CRC liver metastasis xenograft model (86). Finally, the ECM changes, such as excessive fibrosis, can reduce the perfusion of tumor tissue and the efficiency of drug delivery by constricting the tumor blood vessels. The placental growth factor (PIGF) contributes to fibrosis and tissue stiffness in HCC, leading to the development of drug resistance (87). The heterogeneous distribution of abnormal blood vessels is a common feature of solid TME and a major cause of tumor hypoxia. Radiotherapy relies on the action of reactive oxygen species to kill cancer cells, and hypoxia restricts the efficacy of radiotherapy and induces resistance, leading to poor clinical outcomes (88).

### 3.3 Tumor metastasis

Metastasis is a major issue in current cancer treatment, and the majority of cancer patients die from metastatic disease than from primary tumors. Tumor cells penetrate the basement membrane and invade deeper tissue layers during metastasis. This is followed by intravasation into proximal vessels or lymphatics and ultimately extravasation to distant organs by transepithelial migration and capillary rupture. Finally, tumor cells migrate along neurons or directly diffuse into adjacent spaces (89). Unlike those in normal tissue, tumor blood vessels—characterized by immaturity, lack of connection between parietal cells, and high permeability—cannot prevent the dissemination of tumor cells. Matrix metalloproteinases (MMPs) are the important regulators of the TME. They play a unique role in tumor angiogenesis and regulate vascular stability and permeability. MMPs can hydrolyze type IV collagen to increase the permeability of blood vessels and promote tumor invasion. In addition, MMPs can induce the secretion of VEGF by tumor cells to increase the permeability of blood vessels and remodel the ECM (90–93). However, the role of MMPs in cancer is complex. MMP-2 secreted by ovarian tumor cells can regulate their attachment to the peritoneum for metastasis (94). This complexity may be one of the reasons for the failure of multiple clinical trials involving small-molecule MMP inhibitors for cancer treatment (91). In addition, vasculogenic mimicry is one of the manifestations of an abnormal tumor vascular network in which highly aggressive tumor cells replace endothelial cells. This abnormal network not only complements the traditional angiogenic pattern but also plays an important role in tumor metastasis. Vasculogenic mimicry induced by the miR-124/Foxq1/EGFR axis promotes metastasis in nasopharyngeal carcinoma. Anti-EGFR treatment can inhibit the formation of this abnormal vascular network and consequently tumor growth and metastasis (95). Therefore, targeting EGFR signaling can improve the prognosis of metastatic CRC (96).

### 3.4 Ferroptosis

Ferroptosis is a unique mode of cell death driven by iron-dependent phospholipid peroxidation, and resistance to ferroptosis is related to tumorigenesis (97). In addition, ferroptosis is related to the formation of abnormal blood vessels in tumors. The hypoxia in the TME caused by abnormal vascular structure and function is associated with ferroptosis resistance, resulting in tumor development. A hypoxia-induced lncRNA, cystathionine beta-synthase mRNA-transgenic lncRNA, protects gastric cancer cells from ferroptosis, leading to chemoresistance (98). Aberrant expression of various angiogenic factors and receptors caused by abnormal blood vessels inhibits ferroptosis. For example, upregulation of FGFR4 confers anti-HER2 resistance by attenuating ferroptosis in breast cancer. The ANGPTL4 protein, a decisive regulator of angiogenesis, can inhibit ferroptosis to induce resistance against radiation therapy (99, 100). Angiogenic drugs, such as apatinib and sorafenib, sensitize tumor cells to ferroptosis (101, 102). However, ferroptosis has a two-sided effect on tumor progression, and the induction of ferroptosis is sufficient to convert non-suppressive polymorphonuclear-MDSCs (PMN-MDSCs) into immunosuppressive ones. Moreover, the immunosuppressive molecules released by PMN-MDSCs undergoing ferroptosis have an inhibitory effect on T cells (103). Therefore, ferroptosis can lead to tumor immunosuppression. Mouse models with conditional deletion of ferroptosis suppressor genes, such as GPX4 and SLC7A11, accelerated pancreatitis and pancreatic tumorigenesis. The products of oxidative DNA damage during ferroptosis can increase the infiltration of macrophages into pancreatic ductal adenocarcinoma (PDAC), and ferroptotic PDAC cells can release the KRASG12D protein, which promotes macrophage polarization into the M2 phenotype. Notably, inflammation-related immunosuppression due to cell death has the potential to promote tumorigenesis (104).

## 4 TME leads to abnormal vasculature

**Figure 2:**
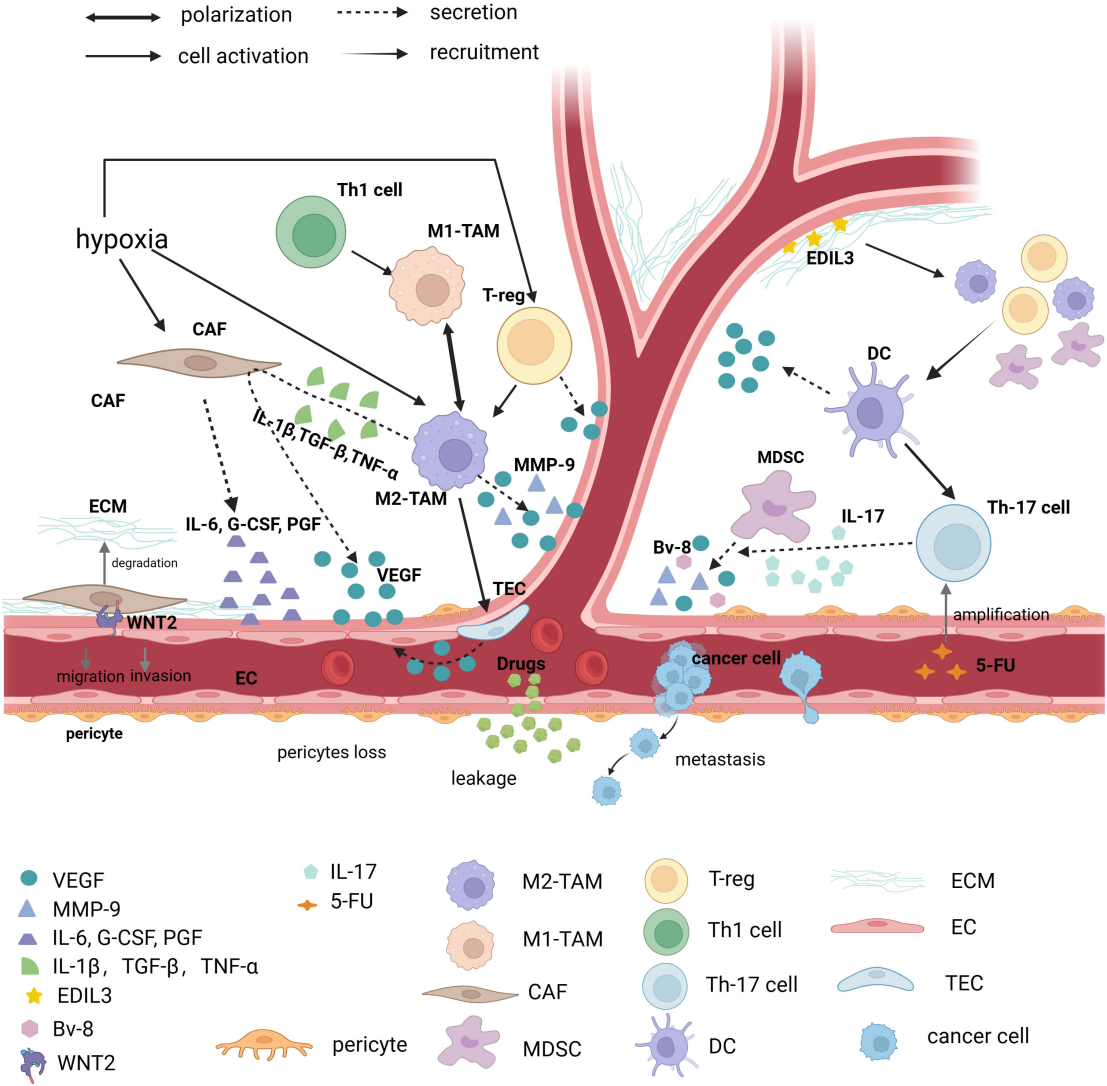
Immunosuppressed TME causes abnormal vessels. Tumor vessels are in an abnormal state of TME (Tumor microenvironment), and various factors that cause immunosuppression such as immune cells, CAF (Cancer associated fibroblasts), ECM (Extracellular matrix), and various immune factors contribute to tumor vessel abnormalities. TAM (Tumor-associated macrophage), and in particular TAM of the M2 phenotype, promotes angiogenesis in at least three ways. First, it directly secretes factors that cause vascular abnormalities, such as: VEGF-A (Vascular endothelial growth factor-A), EGF (Epidermal growth factor), MMP-9 (Matrix metalloproteinase-9), etc., secondly activate tumor endothelial cells to secrete VEGF for angiogenesis, and finally M2-type TAM stimulates CAF to secrete VEGF through IL-1β (Interleukin-1β), TGF-β (Transforming growth factor-β) and TNF-α (Tumor necrosis factor-α). CAF, one of the focuses of TME research, regulates angiogenesis by secreting IL-6, G-CSF (Granulocyte colony-stimulating factor) and PGF (Placental growth factor), and is involved in ECM degradation. Recent studies have found that direct contact of CAF with endothelial cells via WNT2 leads to endothelial cell migration. Other immune cells such as MDSC (Myeloid-derived suppressor cell) secrete VEGF, BV-8, and MMP-9. It is worth noting that Th17 (T helper cell 17) cells can promote MDSC progenitor function through IL-17. Moreover, it has been found that 5-FU can induce immunosuppressive microenvironment by promoting Th17 proliferation via caspase-1. The vascular regulatory function of a T cell depends on its molecular type. Th1 cells can polarize Tams towards the M1 phenotype, but Treg cells drive Tams towards the M2 phenotype and secrete VEGF to promote angiogenesis. DCs (Dendritic cells) are able to secrete VEGF directly, but it can also recruit Treg, MDSC, and M2 macrophages, which exacerbate the immunosuppressive effects of TME. In addition, as one of the important components of TME, ECM can secrete EDIL3 to promote angiogenesis. (The figure was designed in Biorender.com).

The TME includes tumor cells, surrounding non-tumor cells, the secretory products of corresponding cells, and other cellular and non-cellular components of the ECM, such as stromal cells, fibroblasts, and immune cells (105). The progression of the tumor indicates that the TME has the characteristics of immunosuppression. This feature is closely related to the dysfunction of the adaptive immune system, the diversity and plasticity of myeloid cells, and the remodeling of cancer-associated fibroblasts (CAFs) and ECM (106, 107). CD4^+^-deficient mice lack the coverage of pericytes, suggesting that the immunosuppressive TME may have a significant effect on the formation of abnormal tumor vessels (**Figure 2**) (108). Several researchers have proposed the concept of “immunoediting.” Cancer immunoediting has three stages, namely elimination, equilibrium, and escape. The immune system recognizes and kills tumor cells in the elimination phase. Only a few tumor subclones progress to the equilibrium phase, where tumor growth can also be limited or even eliminated by a normal immune system. However, “immunoedited” tumor cell subclones that can evade immune recognition and destruction may enter the escape phase, in which their growth is not restricted and immunosuppressive properties are apparent. The vascular abnormalities are exacerbated due to the appearance of immunosuppressive TME in the immune escape phase (109).

### 4.1 Tumor-associated macrophages

Macrophages are an important component of innate immunity. These cells not only promote inflammation and destroy pathogens but also play a significant role in tissue repair and neovascularization (110, 111). TAMs are strongly associated with an immunosuppressive TME (106). TAMs promote tumor cell proliferation and play a role in cancer progression in sustained cell cultures (112). M0 macrophages differentiate from monocytes under the influence of macrophage colony-stimulating factors. Lipopolysaccharides and Th1-derived cytokines promote the conversion of M0 to M1 macrophages, and IL-4, IL-10, IL-13, IL-33, and TGF-β induce the conversion of M0 to M2 macrophages (113). Although this simplistic classification does not describe the different states of macrophages within tumors, it is commonly assumed that M1 and M2 macrophages can inhibit and promote tumor growth, respectively (114). Macrophages secrete growth factors, such as epidermal growth factor and VEGF; therefore, abnormal blood vessels formed by macrophage activity may be a vital reason for tumor progression in breast cancer (115–117).

M2 macrophages are involved in tumor angiogenesis and invasion (118). Ultrasound-targeted microbubble destruction (UTMD) inhibits the growth and metastasis of pancreatic cancer by regulating vascular normalization. UTMD-induced vascular normalization depends on the polarization of macrophages from the M2 to M1 phenotype (119), indicating that the polarization of TAMs is critical to vascular normalization. Macrophages in injured tissues secrete cytokines, such as insulin-like growth factor-1, VEGFA, and TGF-β, which directly or indirectly promote angiogenesis (120, 121). Similarly, hypoxic TME-induced TAMs can secrete VEGFA and MMP-9 to support angiogenesis (122). In addition, TAMs can promote VEGFA expression in endothelial cells *in vitro (* 123). Therefore, the secretion of pro-angiogenic factors in the TME is associated with TAMs. The M2 polarization of TAMs in phosphatase and tensin homolog-silenced esophageal cancer enhanced the malignant behavior of tumor-associated vascular ECs to form abnormal blood vessels through the PI3K/ATK-dependent pathway (124). TNF-α, secreted by TAMs, upregulated the expression of MALR, an lncRNA, which stabilized the HIF-1α mRNA in esophageal squamous cell carcinoma to promote angiogenesis (125).

A specific classification of TAM surface markers will help understand their role in the TME. The F4/80^+^CD115^+^C3AR^+^CD88^+^ TAMs show a high heme oxygenase-1 (a stress-responsive enzyme) activity, which plays a critical role in shaping a prometastatic TME to favor immunosuppression, angiogenesis, and epithelial-to-mesenchymal transition (126). A subset of TAMs that expressed podoplanin showed high collagenolytic and gelatinase activity, and these TAMs mediated the destruction of the vascular basement membrane to increase vascular permeability. These macrophages directly acted on blood vessels to affect EC junctions, leading to intravasation of tumor cells into blood vessels (127). Tie2-expressing macrophages functioned as a key factor in vascular remodeling after chemotherapy. However, Tie2 expression on macrophages is not effective for tumor angiogenesis. Therefore, additional research is needed to target this phenotype of macrophages for tumor treatment (128, 129).

### 4.2 MDSCs

MDSCs show immunosuppressive activity, and their three recognized subsets are granulocytic/PMN, monocytic, and immature/early MDSCs (130, 131). These cells mainly promote and maintain tumor angiogenesis by secreting MMPs. Mechanistically, MMP-9 promotes angiogenesis and stimulate tumor neovascularization by increasing the bioavailability of VEGF (132). Moreover, VEGF can trigger the recruitment of MDSCs. BV8, highly expressed in MDSCs, promotes angiogenesis and enhances MDSC mobilization in tumors (133). The use of JAK2/STAT3 inhibitors inhibited angiogenesis and decreased the MDSC population in the TME of head and neck carcinoma by inhibiting VEGFA and CK2 (134, 135). Notably, MDSCs isolated 24 h after lung cancer surgery were more potent in promoting angiogenesis and tumor growth than pre-surgery MDSCs, and the number of pulmonary metastatic tumors and MDSCs were positively related to the extent of surgical manipulation (136).

### 4.3 DCs

DCs are antigen-presenting cells that play a crucial role in bridging innate and adaptive immune responses. DCs act as professional antigen-presenting cells to process an antigen and present it to naive T lymphocytes (137). DCs are classified as conventional or classical, plasmacytoid, and monocyte-derived DCs (138). However, antigen presentation is disrupted by the large amount of immature DCs in the TME. Tumor-associated DCs produce IL-23, which can induce IL-17 secretion by Th17 cells to indirectly induce angiogenesis. The newly formed abnormal vessels promote the accumulation of MDSCs and further inhibit the maturity of DCs (139). The classical DCs produce bioactive pro-angiogenic factors, such as VEGFA, FGF2, and ET-1. In addition, DCs can express several chemokines, including CXCL8 and CCL2, which can induce angiogenesis by directly acting on ECs. In addition, CXCL1, CXCL2, CXCL3, and CXCL5 exert an indirect pro-angiogenic effect by recruiting other pro-angiogenic bone marrow cells, including neutrophils (140–142).

### 4.4 T lymphocytes

T lymphocytes are one of the most critical players in adaptive immunity. The effect of T cells on angiogenesis depends on their subtypes and their cytokine profiles. CD4^+^ T lymphocyte deficiency leads to transcriptomic alterations in tumor-associated vascular ECs, resulting in modifications in the pathways or genes known to regulate vascular normalization. Examples of these modifications include the increased expression of VEGFA, decreased expression of Angpt1/Angpt2, and the downregulation of adhesion and ECM molecules (108). CTLs and CD4^+^ Th1 cells can secrete IFN-γ, which restricts the proliferation of ECs and induces their apoptosis, resulting in the restriction of blood flow in the tumor (143). Interestingly, CD8^+^ T lymphocytes exude from blood vessels by regulating the CXCR3/CXCL10 axis, migrate to the retinal tissue, and secrete several factors, such as TNF, IFN-γ, perforin, and granzyme A/B, to promote angiogenesis (144). Moreover, T cells can also affect blood vessels by regulating TAMs in the TME. The IL-8-producing CD4^+^ T lymphocytes in glioma can recruit TAMs and induce their M2 polarization. Notably, blocking IL-8 transforms the M2 TAMs into anti-angiogenic macrophages (145–147). CD4^+^ T lymphocytes, such as Th17 cells and γδT cells, secrete IL-22, which can promote angiogenesis through the STAT3 and MAPK pathways (148, 149).

IL-17-secreting CD4^+^ Th17 cells directly promote angiogenesis and modulate MDSCs to promote angiogenesis independent of VEGF (150). 5-Fluorouracil, an extensively used chemotherapeutic agent, is toxic to MDSCs in the TME. However, 5-fluorouracil activates caspase-1, which could expand the Th17 population. Th17 cells can counter the effects of anti-angiogenic therapy by stimulating neovascularization (151). Hypoxic TME can induce the expression of the chemokine CCL28, leading to the accumulation of Tregs, which limit tumor immunity and promote angiogenesis. Mechanistically, miRNA21 can induce the ICOS expression on Tregs, and the crosstalk of ICOS ligands with Tregs can activate ECs to induce abnormal angiogenesis (152–154).

### 4.5 Cancer-associated fibroblasts

CAFs play a key role in the TME and affect the malignant progression of tumors through multiple processes, including remodeling of ECM, increasing the production of growth factors, and promoting angiogenesis (155). Bioinformatic analysis has demonstrated that CAFs can be considered prognostic markers in certain tumors. Higher stromal/CAF scores were associated with poor overall survival in patients with ovarian cancer and poor immune response to treatment (156). CAF-derived wingless-type MMTV integration site family member 2 (WNT2) increased tumor angiogenesis in colon cancer, and its overexpression increased blood vessel density and tumor volume in CRC xenografts. Unterleuthner, D. et al. analyzed publicly available datasets for human CRC and found decreased survival rates in patients diagnosed with tumors having high WNT2 expression (157). Furthermore, IL-6 mediates the crosstalk between tumor cells and CAFs by supporting tumor growth and promoting fibroblast activation. Athymic nude mice with head and neck squamous cell carcinoma patient-derived xenografts (PDX) and gastric adenocarcinoma PDX were treated with tocilizumab, an anti-IL6Rα antibody that inhibits tumor growth *in vivo* in part by inhibiting the STAT3 and MEK/ERK signaling. The treatment induced tumor growth arrest and reduced STAT3 and ERK1/2 signaling (158). The analysis of phosphorylated STAT3 expression in CAFs of human tissue microarrays demonstrated a negative correlation between its increased stromal expression and the survival of patients with CRC. Mechanically, STAT3 activation plays an essential role in the development of CAF-mediated CRC. Therefore, STAT3 activation increases the expression of several angiogenic signals, thereby promoting CRC progression (159). CAFs can mediate the tumor vascular abnormalities caused by hypoxia. They increase the secretion of the hypoxia-induced angiogenesis regulator NCBP2-AS2 (the uncharacterized protein renamed from hypoxia-induced angiogenesis regulator), thereby enhancing the VEGF signaling to promote endothelial sprouting (160).

### 4.6 Extracellular matrix

ECM is a complex and dynamic structure composed of macromolecular substances secreted by cells into the extracellular space comprising interstitial matrix and basement membrane. In addition, ECM contains various secreted proteins, including cytokines, chemokines, and growth factors, involved in immune cell regulation. Collagen is one of the main components of the ECM, and high-density collagen can guide macrophages to acquire an immunosuppressive phenotype (161). Therefore, ECM is a highly dynamic partner of the immune system (162, 163). Epidermal growth factor-like repeats and discoidin I-like domains 3 (EDIL3), an ECM protein highly expressed in HCC, contributes to angiogenesis. Moreover, autocrine EDIL3 can support tumorigenesis by promoting resistance to anoikis in HCC cells (164). Notably, VEGF induces high EDIL3 expression in malignant cells, suggesting that high VEGF in an immunosuppressive microenvironment can indirectly affect tumor blood vessels by changing the composition of ECM (165, 166).

## 5 Vascular normalization and immunotherapy

​Solid tumors require blood vessels to grow, and many new cancer therapies target tumor blood vessels. Traditional anti-angiogenic “vessel blocking” strategies attempt to inhibit new blood vessel formation and destroy existing blood vessels to cause nutrient starvation in tumors. However, their success is limited by inadequate efficacy or the development of drug resistance. In recent years, the concept of “vascular normalization” has been applied to cancer treatment. Some anti-angiogenic agents can temporarily “normalize” the abnormal structure and function of the tumor vasculature, making it more efficient for oxygen and drug delivery. The appropriate use of drugs that induce vascular normalization can alleviate hypoxia and improve the efficacy of conventional therapies (167, 168).

The inhibition and reprogramming of the immune system play a key role in the occurrence and development of tumors. Immunotherapy aims to reactivate antitumor immune cells and overcome the immune escape mechanism in the tumors (109). The discovery of IL-2, T-cell dual signal activation pathway, CTLA-4, PD-1, and several other immune molecules led to the concept of “immune checkpoint blockade” in tumor treatment. However, all patients do not respond to these therapies, indicating the complexity of tumor-induced immune alterations. Therefore, finding a synergistic method to activate anti-tumor T cell responses and target inhibitory TME has been a major research focus in tumor immunotherapy (169). The crosstalk between vascular abnormalities and immunosuppression suggests that a combination of the two approaches would have a greater therapeutic effect.

### 5.1 Vascular normalization strategies

#### 5.1.1 Targeting the VEGF/VEGFR pathway

VEGFA, VEGFB, and PIGF are primarily involved in the regulation of angiogenesis and vascular permeability. These molecules bind to VEGFR-1, which is primarily located on the surface of vascular endothelial cells. However, the primary function of VEGFA is accomplished by binding to VEGFR-2. Neuropilins-1 and -2 act as co-receptors for the VEGF ligands (53, 170). VEGFA induces the VEGFR-2 dimerization and triggers its autophosphorylation of VEGFR-2 to activate the downstream signal transduction pathways, including the PI3K, PLC-γ, Akt, Ras, and MAPK pathways, to promote cell proliferation, survival, migration, permeability, and differentiation and regulate cell adhesion molecules in addition to other functions (171, 172). VEGFs regulate EC proliferation, increase vascular permeability, and aggravate immunosuppression. Drugs targeting the VEGF/VEGFR pathway can achieve vascular normalization by inhibiting these functions. Bevacizumab (a recombinant humanized monoclonal antibody) was the first clinically approved drug, which increased the function of other cytotoxic agents through vascular normalization. Notably, vessel diameter, density, permeability, and interstitial fluid pressure decreased after the treatment (173).

Tyrosine kinase inhibitors (TKIs), such as apatinib, cedianib, and anlotinib, are being used to induce vascular normalization in tumors. TKIs inhibit the catalytic domain of receptor tyrosine kinases in a competitive or allosteric manner. Specifically, apatinib targets VEGFR-2, anlotinib targets VEGFR, PDGFR, and FGFR, and desinib targets VEGFR-1/2/3, PDGFR-α, CSF-1R, and Flt3. Notably, TKIs have more targets than macromolecular monoclonal antibodies. The combination of TKIs and cytotoxic drugs is one of the active research areas in cancer therapeutics. Wang, T. et al. conducted a randomized clinical trial based on the effect of apatinib plus pegylated liposomal doxorubicin (PLD) versus PLD alone in patients with platinum-resistant recurrent ovarian cancer. The combination treatment showed a promising efficacy with manageable toxic effects. Apatinib plus PLD group has improved median progression-free survival (5.8 months) compared with the PLD group (3.3 months). The median overall survival was 23.0 months in the apatinib plus PLD group compared with 14.4 months in the PLD group. The disease control rate was 81.5% in the apatinib plus PLD group and 53.1% in the PLD group (174). However, patients receiving combination therapy with cedeinib and cytotoxic drugs for glioblastoma showed improvements in OS only in the subgroup with increased tumor perfusion and oxygenation. The investigators suggested that this phenomenon was related to vascular normalization after combination therapy. In addition, this finding explains the failure of sildenib and bevacizumab therapy to prolong overall survival in clinical studies (175, 176). Therefore, further research is required to select appropriate TKIs for cancer treatment.

Moreover, anti-VEGF therapy could improve the delivery and efficacy of CAR-T therapy. Combination treatment with an anti-mouse VEGF antibody improved the CAR-T cell infiltration and distribution throughout the TME, delayed tumor growth, and improved the ability of CAR-T cells to penetrate and distribute throughout the TME compared with CAR-T-cell therapy alone. In addition, the combination therapy prolonged the survival of tumor-bearing mice (177). Other therapies, such as bispecific antibodies, have shown promising antitumor activity in experimental studies. Combined blockade of ANGPT2 and VEGFA with a bispecific antibody (A2V) showed superior therapeutic efficacy compared with single agents in genetically engineered and transplanted tumor models, including metastatic breast cancer, pancreatic neuroendocrine tumors, and melanoma models. Notably, this therapeutic effect is related to immune function (178).

Th1 cells in the TME play a crucial role in vascular normalization. The disruption of vascular normalization reduced the T-lymphocyte infiltration and depletion, or inactivation of CD4^+^ T lymphocytes reduced vascular normalization, suggesting a reciprocal regulatory cycle (108). Anti-angiogenic immune-modulating therapy affects the lymphotoxin/lymphotoxin beta-receptor (LT/LTβR) signaling pathway through CD8^+^ T and NK cell signaling and eventually induces the differentiation of postcapillary venules into inflamed high endothelial venules (HEVs). Tumor HEVs enhance the proliferation of TCF-1^+^PD-1^+^ lymphocytes and the production of cytotoxic PD-1^+^TIM-3^+^ lymphocytes by changing the perivascular microenvironment to promote antitumor immunity (179). Therefore, the combination of anti-VEGF/VEGFR and immunotherapy can enhance tumor immunity by multiple ways, such as relieving the inhibition of antigen presentation and inhibiting the recruitment of immunosuppressive cells.

#### 5.1.2 Blocking other alternative targets

Angiopoietins include ANG1, ANG2, and ANG4, and their receptors include Tie1 and Tie2. ANG1 can tightly bind Tie2 at nanomolar affinity. This binding can reduce angiogenesis and vascular permeability, promote endothelial cell maturation, favor vascular stabilization, and modulate vascular normalization during anti-angiogenic therapy. ANG2 is a partially competitive antagonist of ANG1 for binding to Tie2. ANG2 prevents the phosphorylation of Tie2 by ANG1. However, when ANG1 is absent, ANG2 can replace ANG1 and binds to Tie2. The level of ANG2 increases under inflammatory and hypoxic conditions, which reduces vascular stability and enhances EC activation, angiogenesis, and remodeling (180). Therefore, neutralizing ANG2 by preventing its binding to Tie2 is the main research direction for developing drugs targeting the ANG–Tie pathway. However, multiple clinical trials of monoclonal antibodies against ANGs have been unsuccessful. A phase I clinical study on MEDI3617, a monoclonal antibody against ANG2, showed long-term grade 3 edema-related adverse events in patients, and the drug development was terminated due to its limited clinical activity. Nesvacumab was also discontinued because no significant benefit was found with reference to the comparison arm of the experimental therapy (181, 182). Moreover, resistance to classical anti-VEGF agents inevitably emerges, and the most common mechanism involves increased tumor hypoxia levels induced by anti-angiogenic therapy (183). A hypoxic environment induces the upregulation of other angiogenic factors, such as ANG2, FGFR, and EGFR (184). Ectopic expression of ANG2 reduces the beneficial effects of VEGFR-2 blockade by inhibiting vascular normalization. Therefore, simultaneous blocking of these angiogenic pathways (7, 185) will increase the efficacy of the treatment (178, 186).

FGFs are angiogenic factors that stimulate vascular cell proliferation, migration, and differentiation (187). Upregulation of FGF has been associated with tumor resistance to anti-VEGF therapy (188), which illustrates that combining FGF/FGFR inhibition with anti-VEGF therapy can lead to higher antitumor efficacy. Infigratinib, a pan-FGFR inhibitor, targets FGFR to inhibit cell proliferation, angiogenesis rescue programs, hypoxia, invasion, and metastasis (189). FGF401, another agent targeted on FGFR4, selectively improves the chemotherapy outcomes in mice bearing high FGF19-expressing HCC tumors (190). Combining anti-VEGF agents with the inhibition of the receptor kinase of VEGF, FGF, and PDGF can synergistically inhibit tumor growth and enhance response to radiation therapy (191).

PDGF/PDGFR is one of the important targets, and PDGF-B and PDGFR-β are being actively explored. However, the effect of tumor PDGF-B on vascular maturity has been controversial (192). PDGFR-β is required for the recruitment of pericytes to tumor blood vessels, and EC-derived PDGF-B is essential for the proper integration of pericytes in the vessel wall (193). PDGF-B can stabilize blood vessels; however, its high expression in tumors is inconsistent with the occurrence of numerous immature blood vessels in tumors (194, 195). The above-threshold level of PDGF-BB, such as high PDGF-B expression in metastatic breast cancer can induce pericyte loss and promote vascular leakage. On the contrary, low PDGF-B expression was found in metformin-induced vascular normalization. The blockade of PDGF-B/PDGFR-β significantly enhances vascular maturity in tumors with high PDGF-B expression, whereas an opposite effect is observed in tumors with low PDGF-B expression (194, 196). Therefore, further studies are required to define the optimal conditions and the amount of PDGF-B inhibitors for vascular normalization.

### 5.2 Immunotherapy interacts with vascular normalization

#### 5.2.1 Mechanism of reciprocal inhibition

A fundamental mechanism of vascular normalization in synergy with immunotherapy is to increase the effective immune response by increasing the number of immunostimulatory cells or decreasing the immunosuppressive cells. Combined anti-VEGF and immune checkpoint blockade treatment increased M1-TAM subpopulations in HCC. The infiltration and activation of cytotoxic T cells increased, whereas the infiltration of Tregs and CCR2^+^ monocytes decreased after combination therapy (197). Compared with nintedanib monotherapy for lung cancer, combined therapy with αPD-L1 was more effective in promoting the infiltration of activated T cells and DCs and eliminating the immunosuppressive environment by reducing the proportion of MDSCs and TAMs (198). Combination therapy can improve the efficacy of immunotherapy by relieving high interstitial pressure, increasing the infiltration of immune cells, enhancing the response to IFN-γ, and upregulating the MHC-I expression in tumor cells (198). In addition, the endothelial ICAM-1-induced transendothelial migration of leukocytes increased the infiltration of immune cells after vascular normalization. Antigenic compounds induce the expression of endothelial ICAM-1, implying that VEGF-targeted therapy can counteract tumor endothelial cell anergy and promote the formation of inflammatory infiltrates in tumors (49). Moreover, Rac1 and its effector molecules, such as Trio, Tiam, and Rhog, may contribute to the increased trans-endothelial migration of T cells. Rac1 and Rhog are the members of the rho-GTPase family. Tiam and Trio are the guanine nucleotide exchange factors, which stimulate the release of GDP and promote GTP binding. Rac1, widely expressed in tissues, is a regulator associated with cell motility and invasion (199). Myct1, a direct target gene of ETV2, is involved in regulating the angiogenic function of ECs, and more CD8^+^ T cells can migrate through the Myct1-deficient EC barrier. The expression of Rac1 was increased in Myct1-deficient ECs in tumors, and inhibition of Rac1 abolished the increase in T-cell migratory phenotype (200). Finally, the formation of HEVs (the blood vessels usually found in secondary lymphoid tissues) promotes the infiltration of lymphocytes. HEV promotes the infiltration of CD8^+^ CTLs in solid tumors, and their presence is associated with slower tumor growth and better prognosis in patients (201). Combined anti-VEGFR2 therapy with anti-PD-L1 antibodies induced HEV in breast cancer and pancreatic neuroendocrine tumors (202).

Moreover, VEGFR-2 blockade alone increased the expression of PD-1 in tumor-infiltrating CD4^+^ cells in an endothelial IFN-γ dependent manner (197). This suggests that the combined therapy with PD-1 can reduce the negative effects of the anti-VEGF monotherapy. In addition, combination therapy increases the density of DCs and promotes their activation (198). This change indirectly restores the function of suppressed T cells, a goal that cannot be achieved with either drug alone. In addition, targeting peroxisome proliferator-activated receptor-gamma, a factor that activates VEGFA transcription, can avoid T-cell dysfunction caused by T-cell exhaustion (203).

Compared with anti-VEGFR-2 therapy alone, dual anti-PD-1/VEGFR-2 therapy can promote CD4^+^ T cell-induced vascular normalization, indicated by the increased density of microvessels covered by pericytes and the protection against increased hypoxia in HCC (197). The blocking of TGF-β1 signaling favors the proliferation and expression of adhesion molecules, such as E-selectin in ECs, leading to the densification and normalization of the vasculature within the tumors. Moreover, the co-blockade of TGF-β1/PD-1 increased the density of blood vessels covered by pericytes (204). CD4^+^ and CD8^+^ T cells effectively mediate vascular normalization in breast cancer models (108, 205). Therefore, the concepts of vascular normalization and immunotherapy are combined to avoid excessive vascular pruning caused by the use of anti-angiogenic drugs and normalize the tumor vascular network. The normalized blood vessels can stimulate the infiltration of immune cells and enhance the efficacy of immunotherapy. Simultaneously, an immune-stimulated TME can promote tumor vascular normalization; therefore, a virtuous circle is formed between vascular normalization and immunotherapy.

#### 5.2.2 Regimens of combined therapy

Anti-angiogenic agents synergize with immune checkpoint blockade; however, the sequence, dose, and timing of administration remain to be studied. Huang, Y. et al. suggested that a normalization “window” exists, depending on the timing and dose of anti-angiogenic therapy (206). Li, Q. et al. showed that both standard and low-dose anti-angiogenic drugs had vascular normalization effects in breast cancer. However, low-dose anti-angiogenic drugs were more effective in promoting the activated immune cell infiltration and PD-1 expression compared with standard dose drugs. Clinical studies on patients with advanced triple-negative breast cancer revealed that the combination of low-dose apatinib and immune checkpoint blockade shows better efficacy and tolerance (207). Given the reciprocal mechanisms, the sequence of anti-angiogenic and immunotherapy treatments is critical. The ideal effect can be achieved by using anti-angiogenic drugs to mediate vascular normalization before immune checkpoint blockade treatment. The efficacy and mechanism of immunotherapy followed by anti-angiogenic therapy have been explored. Administration of sorafenib, an inhibitor of the pan-vascular endothelial growth factor receptor, improved outcomes in patients with HCC after initial treatment with anti-PD-1 antibody. In addition, the infiltration of immune cells and the degree of vascular normalization were improved (208). This study specified the order of drug use but failed to compare the tested order with other orders of drug use; therefore, a further expansion of this concept is needed.

### 5.3 Clinical trials of combination therapy

Combined therapy includes anti-angiogenic drugs, such as large-molecule monoclonal antibodies and small-molecule TKIs, for vascular normalization and immune checkpoint inhibitors for immunotherapy in clinical trials. In addition, bispecific antibodies, such as PD-L1 and VEGF dual fusion antibodies, have also been used for combination therapy. Several combination regimens have already been approved by the FDA, and a large number of therapies are being tested (**Table 1**).

**Table 1:** Clinical trials based on vascular normalization and immunotherapy.

Interventions	Diseases	Phases	NCT Number
PM8002(Anti-PD-L1/VEGF)+ Platinum + Atezolizumab+ Etoposide	SCLC	II&III	NCT05844150
Surufatinib + KN046(Anti-PD-L1/CTLA-4) + Gemcitabine + Nab paclitaxel	PC	I&II	NCT05832892
B1962(PD-L1/VEGF bispecific antibody fusion protein)	Neoplasms Malignant	I	NCT05650385
FOLFOX + Balstilimab + Bevacizumab + Botensilimab	CRC	I&II	NCT05627635
Pembrolizumab + Lenvatinib	Melanoma	II	NCT05545969
Atezolizumab+ Bevacizumab	Neoplasms Malignant	III	NCT05482516
Lenvatinib+ Pembrolizumab+ Etoposide+ Carboplatin	SCLC	II	NCT05384015
Bevacizumab + Camrelizumab	Malignant Pleural Effusion	I&II	NCT05330065
Durvalumab + Lenvatinib	HCC	II	NCT05312216
Camrelizumab + Apatinib	HNSCC	II	NCT05156970
mFOLFOX6 + Atezolizumab + Bevacizumab	BTC	I&II	NCT05052099
AK112(PD-1/VEGF Bispecific Antibody)	NSCLC	I&II	NCT04900363
Atezolizumab + Bevacizumab + Gemcitabine + Carboplatin	BC	II	NCT04739670
Axitinib + Avelumab	RCC	II	NCT04698213
Atezolizumab+ Bevacizumab	HCC|NSCLC	II	NCT04563338
Pembrolizumab + Lenvatinib	BTC	II	NCT04550624
Camrelizumab+ Apatinib	NPC	II	NCT04548271
Camrelizumab+ Apatinib	NPC	II	NCT04547088
Atezolizumab+ Bevacizumab	OC|FTC|PC	II	NCT04510584
Camrelizumab+ Lenvatinib	HCC	I&II	NCT04443309
Atezolizumab+ Bevacizumab	Melanoma	II	NCT04356729
Docetaxel+ Pembrolizumab+ Ramucirumab	NSCLC	II	NCT04340882
Pembrolizumab+ Lenvatinib	Melanoma	II	NCT04207086
Regorafenib+ Nivolumab	HCC	I&II	NCT04170556
Axitinib + Toripalimab	HCC|BTC	II	NCT04010071
Durvalumab + Cediranib	CRCr|PAAD|Leiomyosarcoma	II	NCT03851614
Rucaparib, Abemaciclib, pembrolizumab + bemcentinib, Atezolizumab + Bevacizumab, Dostarlimab + Niraparib	Mesothelioma, Malignant	II	NCT03654833
Niraparib+ Dostarlimab +Standard of care(paclitaxel + carboplatin + bevacizumab)	ON|PC	III	NCT03602859
Atezolizumab+ Cobimetinib+ Ipatasertib+ Bevacizumab	BC|BC	II	NCT03395899
Avelumab + Axitinib	HCC	I	NCT03289533
Atezolizumab+ Bevacizumab	Cervical Adenocarcinoma	II	NCT02921269
Durvalumab+ Bevacizumab	BC	Early I	NCT02802098
Axitinib + Pembrolizumab	RCC	I	NCT02133742

RCC, renal cell carcinoma; OC, ovarian cancer; BC, breast cancer; FTC, fallopian tube cancer; NSCLC, non-small cell lung cancer; PC, peritoneal cancer; SCLC, small cell lung cancer; ON, ovarian neoplasms; CRC, colorectal cancer; HCC, hepatocellular carcinoma; HNSCC, head and neck squamous cell carcinoma; BTC, biliary tract carcinoma; NPC, nasopharyngeal carcinoma; PAAD, pancreatic adenocarcinoma.

Vascular normalization has proven to be a successful therapeutic strategy in RCC. The response to chemotherapy is poor, and targeted therapy against VEGFR is used as the first-line treatment in advanced RCC (overall survival: 8–9 months) (209). Several immune checkpoint inhibitors combined with anti-angiogenic drugs have been approved for the treatment of RCC, including atixinib/pembrolizumab, axitinib/avelumab, cabozantinib/nivolumab, and lenvatinib/pembrolizumab (170). The combination of lenvatinib and pembrolizumab leads to a longer overall and progression-free survival compared with sunitinib monotherapy. Moreover, this combination shows certain advantages in progression-free survival over lenvatinib plus everolimus treatment (210). In addition, the addition of atezolizumab to bevacizumab plus chemotherapy significantly improved overall and progression-free survival in patients with metastatic nonsquamous non-small cell lung cancer, irrespective of PD-L1 expression and EGFR or ALK genetic alteration status (211). This treatment was subsequently approved for clinical use. Overall, the concept of combining vascular normalization and immunotherapy is gradually being validated in clinical practice.

## 6 Discussion

All the current drugs available for vascular normalization target angiogenic factors and their upstream/downstream pathways, reflecting the importance of TME in tumor therapy. Moreover, the concept of immune response in the TME has also been extended from the role of immune cells to that of other components, such as blood vessels, ECM, and CAFs. In addition, the relationship between tumor vascular normalization and tumor immunity has also been extended to the entire TME. Here, we summarized the role of CAF, ECM, and cytokines in the cycle of tumor vascular normalization and tumor immunity. The combination of anti-angiogenic drugs and immunotherapy focuses on vascular normalization and provides a new direction for cancer treatment by increasing the efficacy through reciprocal mechanisms and decreasing side effects by reducing the dosage of each drug (7). However, the availability of combination strategies is limited, and numerous combination regimens are only available for highly angiogenic tumors such as RCC. Clinical trials for combination therapy in poorly vascularized tumors, such as PDAC, after the failure of anti-VEGF and immune checkpoint blockade monotherapy are lacking (212–214). It is difficult to achieve the same efficacy for the same therapy for different types of tumors or even the same type of tumors, which denotes the heterogeneity in tumor blood vessels and tumor immunity. Circulating markers, such as serum ANG2, and the ECM molecule EMILIN-2 (a pro-angiogenic molecule) are associated with the therapeutic effect of immune checkpoint blockade (215, 216), indicating that they may be considered new markers for predicting the efficacy of immunotherapy. Changes in vascular function can be directly visualized with imaging studies, such as Doppler ultrasonography, perfusion scanning, or dynamic contrast-enhanced magnetic resonance imaging (217, 218). In conclusion, the close relationship between vascular normalization and tumor immunity determines their potential applications in cancer treatment.

## Author contributions

CQ: Conceptualization, Validation, Writing – original draft. CL: Conceptualization, Validation, Writing – original draft. WL: Data curation, Visualization, Writing – original draft. RZ: Data curation, Visualization, Writing – review & editing. LZ: Methodology, Project administration, Resources, Supervision, Writing – review & editing.
